# Supplementary material for: POLD1 as a Prognostic Biomarker Correlated with Cell Proliferation and Immune Infiltration in Clear Cell Renal Cell Carcinoma
Source: Int J Mol Sci. 2023 Apr 6;24(7):6849. doi: 10.3390/ijms24076849 (PMC10095303; doi:10.3390/ijms24076849)
Supplement: Supplementary file 1 [file ijms-24-06849-s001.zip › Supplementary Figures caption.pdf]

Supplementary Figure S1. Correlation between POLD1 expression and the clinical parameters of ccRCC patients and its prognostic significance in the validation cohort. (A) Relationship of POLD1 mRNA levels with individual cancer stages (T stage, N stage and M stage) and tumor grade of ccRCC patients in the validation cohort. (B) Relationship of POLD1 expression levels with overall survival (OS) in the validation cohort. (C) The AUC for 1-, 3-, and 5-year predicted OS in the validation cohort. (D) Univariate and multivariate survival analyses for selecting prognostic factors on the validation cohort.

Supplementary Figure S2. Association between POLD1 expression and the clinical parameters of localized ccRCC patients and its prognostic significance. (A, B) Univariate and multivariate survival analyses for selecting prognostic factors. (C, D) Establishment (C) and estimate (D) of the overall survival nomogram (3-, 5-, and 8-year) for localized ccRCC patients.

Supplementary Figure S3. Association between POLD1 expression and the clinical multiple categorical variables of ccRCC patients and its prognostic significance. (A-D) Relationship of POLD1 mRNA levels with individual cancer stages (T stage, N stage and M stage) and tumor grade of ccRCC patients. (E, F) Univariate and multivariate survival analyses for selecting prognostic factors. (G, H) Establishment (G) and estimate (H) of the overall survival nomogram (3-, 5-, and 8-year) for ccRCC patients.

Supplementary Figure S4. Mutation feature of POLD1 in ccRCC from TCGA cohort. (A) The alteration frequency with mutation type of POLD1 in different tumor samples from TCGA cohort. (B) Mutation sites of POLD1 in ccRCC from TCGA cohort. (C, D) The mutation patterns of ccRCC patients with high- or low- POLD1 expression were analyzed respectively.

Supplementary Figure S5. Analysis of the activity of infiltrating immune cells in $POLD1^{high}$ and $POLD1^{low}$ groups in ccRCC. (A) Box plot shows differences in

composition of four types of immune cells between $POLD1^{high}$ and $POLD1^{low}$ groups. (B) ssGSEA analysis identifying the relative infiltration of immune cell populations for 541 ccRCC tumor samples with available RNA-sequencing data from TCGA cohort. (C) Difference in the immune checkpoints (PD-1, PD-L1, and CTLA-4) in TCGA cohort stratified by high-/low- POLD1 of KIRC. *** $p < 0.001$.
