# Supplementary material for: POLD1 as a Prognostic Biomarker Correlated with Cell Proliferation and Immune Infiltration in Clear Cell Renal Cell Carcinoma
Source: Int J Mol Sci. 2023 Apr 6;24(7):6849. doi: 10.3390/ijms24076849 (PMC10095303; doi:10.3390/ijms24076849)
Supplement: Supplementary file 1 [file ijms-24-06849-s001.zip › Supplementary Table S1. Clinicopathological characteristics in relation to POLD1 expression level in the TCGA cohort.pdf]

Supplementary Table S1. Clinicopathological characteristics in relation to POLD1 expression level in the TCGA cohort.

Characteristics	Patients	POLD1 expression		χ2	*P* value
		Low (%)	High (%)		
**Age**				0.236	0.627
<= 60	262	128 (48.8%)	134 (51.2%)		
>60	251	129 (51.4%)	122 (48.6%)		
**Gender**				1.858	0.173
Male	337	161 (47.7%)	176 (52.3%)		
Female	176	96 (54.6%)	80 (45.4%)		
**Histologic grade**				10.833	**0.027** [a]
G1	12	5 (41.6%)	7 (58.4%)		
G2	219	121 (55.3%)	98 (44.7%)		
G3	201	99 (49.2%)	102 (50.8%)		
G4	73	26 (35.6%)	47 (64.4%)		
Gx	8	6 (75%)	2 (25%)		
**Pathologic stage**				16.331	**0.001** [a]
Stage I	255	142 (55.7%)	113 (44.3%)		
Stage II	56	28 (50%)	28 (50%)		
Stage III	117	61 (52.2%)	56 (47.8%)		
Stage IV	82	25 (30.4%)	57 (69.6%)		
NA	3	1 (33.3%)	2 (66.7%)		
**T stage**				9.581	**0.022** [a]
T1	261	145 (55.6%)	116 (44.4%)		
T2	68	32 (47%)	36 (52.9%)		
T3	173	78 (45%)	95 (55%)		
T4	11	2 (18.1%)	9 (81.9%)		
**N stage**				4.172	0.132 [a]
N0	229	116 (50.7%)	113 (49.3%)		
N1	16	4 (25%)	12 (75%)		
Nx	268	137 (51.2%)	131 (48.8%)		
**M stage**				35.724	**< 0.001** [a]
M0	407	230 (56.6%)	177 (43.4%)		
M1	78	24 (30.7%)	54 (69.3%)		
Mx	28	3 (10.7%)	25 (89.3%)		
**OS event**				7.082	**0.008**
Alive	334	187 (54.4%)	157(45.6%)		
Dead	169	70 (41.4%)	99 (58.6%)		
**PFS event**				6.775	**0.009**
No	353	191 (54.2%)	162 (45.8%)		
Yes	160	66 (41.2%)	94 (58.8%)		
**DSS event**				14.541	**< 0.001**
No	405	221 (54.6%)	184 (45.4%)		
Yes	108	36 (33.3%)	72 (66.7%)		

Statistical significance was determined by Chi-square test (if necessary, results were adjusted by Yate’s correction) or Fisher's exact test ([a]). OS: Overall survival; PFS: Progression free survival; DSS: Disease specific survival.
