# Supplementary material for: POLD1 as a Prognostic Biomarker Correlated with Cell Proliferation and Immune Infiltration in Clear Cell Renal Cell Carcinoma
Source: Int J Mol Sci. 2023 Apr 6;24(7):6849. doi: 10.3390/ijms24076849 (PMC10095303; doi:10.3390/ijms24076849)
Supplement: Supplementary file 1 [file ijms-24-06849-s001.zip › Supplementary Table S2. Clinicopathological characteristics in relation to POLD1 expression level in the validation cohort.pdf]

Supplementary Table S2. Clinicopathological characteristics in relation to POLD1 expression level in the validation cohort.

Characteristics	Case (N=60)	IHC score < 6 (%)	IHC score >= 6 (%)	χ2	*P* value
**Age**				6.89	0.009[a]
<= 60	38	20 (52.6%)	18 (47.4%)		
>60	22	4 (18.2%)	18 (81.8%)		
**Gender**				0	0.999
Male	35	14 (40.0%)	21 (60.0%)		
Female	25	10 (40.0%)	15 (60.0%)		
**Histologic grade**				1.35	0.245
G1-G2	32	15 (46.9%)	17 (53.1%)		
G3-G4	28	9 (32.1%)	19 (67.9%)		
**T stage**				1.60	0.206
T1-T2	42	19 (45.2%)	23 (54.8%)		
T3-T4	18	5 (27.8%)	13 (72.2%)		
**N stage**				4.94	0.008[a]
N0	51	24 (47.1%)	27 (52.9%)		
N1	9	0 (0%)	9 (100.0%)		
**M stage**				0.91	0.340[a]
M0	55	23 (41.8%)	32 (58.2%)		
M1	5	1 (20.0%)	4 (80.0%)		
**Laterality**				0.19	0.662
Left	22	8 (36.4%)	14 (63.6%)		
Right	38	16 (42.1%)	22 (57.9%)		
**Tumor size**				0.18	0.673
< 4cm	28	12 (42.9%)	16 (57.1%)		
>= 4cm	32	12 (37.5%)	20 (62.5%)		
**Status**				0.40	0.526[a]
Alive	56	23 (41.1%)	33 (58.9%)		
Dead	4	1 (25.0%)	3 (75.0%)		

Statistical significance was determined by Chi-square test (if necessary, results were adjusted by Yate's correction) or Fisher's exact test ([a]). IHC: Immunohistochmeistry.
