# Supplementary material for: POLD1 as a Prognostic Biomarker Correlated with Cell Proliferation and Immune Infiltration in Clear Cell Renal Cell Carcinoma
Source: Int J Mol Sci. 2023 Apr 6;24(7):6849. doi: 10.3390/ijms24076849 (PMC10095303; doi:10.3390/ijms24076849)
Supplement: Supplementary file 1 [file ijms-24-06849-s001.zip › Supplementary Table S3. Correlation analysis between POLD1 and related genes and markers of Treg cell and T cell exhaustion in GEPIA.pdf]

Supplementary Table S2. Correlation analysis between POLD1 and related genes and markers of Treg cell and T cell exhaustion in GEPIA.

Description	Gene markers	Kidney					
		Tumor		Normal		GTEx	
		R	*P*	R	*P*	*R*	*P*
Treg	FOXP3	0.320	3.40E-14*	0.390	7.20E-04*	0.580	1.30E-03*
	CCR8	0.086	5.00E-02	0.093	4.40E-01	0.110	5.70E-01
	STAT5B	-0.190	9.20E-06*	0.320	7.00E-03*	0.760	5.60E-06*
	TGFβ(TGFB1)	0.230	1.90E-07*	0.780	4.80E-16*	0.870	1.10E-06*
T cell exhaustion	PD-1 (PDCD1)	0.340	5.40E-16*	-0.014	9.10E-01	0.550	2.30E-03*
	PDL1(PDCD1LG2)	-0.110	1.00E-02*	0.250	3.60E-02*	0.580	1.20E-03*
	CTLA4	0.290	8.80E-12*	0.100	3.90E-01	0.590	9.80E-04*
	LAG3	0.380	3.40E-19*	0.630	2.40E-09*	0.810	1.90E-07*
	TIM-3 (HAVCR2)	-0.073	9.50E-02	-0.430	1.90E-04*	0.160	4.00E-01
	GZMB	0.030	1.10E-12*	0.0094	9.40E-01	0.091	6.40E-01

GTEx: Genotype-Tissue Expression; R: Value of Spearman's correlation; *P:* Statistical significance. * $P < 0.05$.
