# Supplementary figures and images for: POLD1 as a Prognostic Biomarker Correlated with Cell Proliferation and Immune Infiltration in Clear Cell Renal Cell Carcinoma
Source: Int J Mol Sci. 2023 Apr 6;24(7):6849. doi: 10.3390/ijms24076849 (PMC10095303; doi:10.3390/ijms24076849)

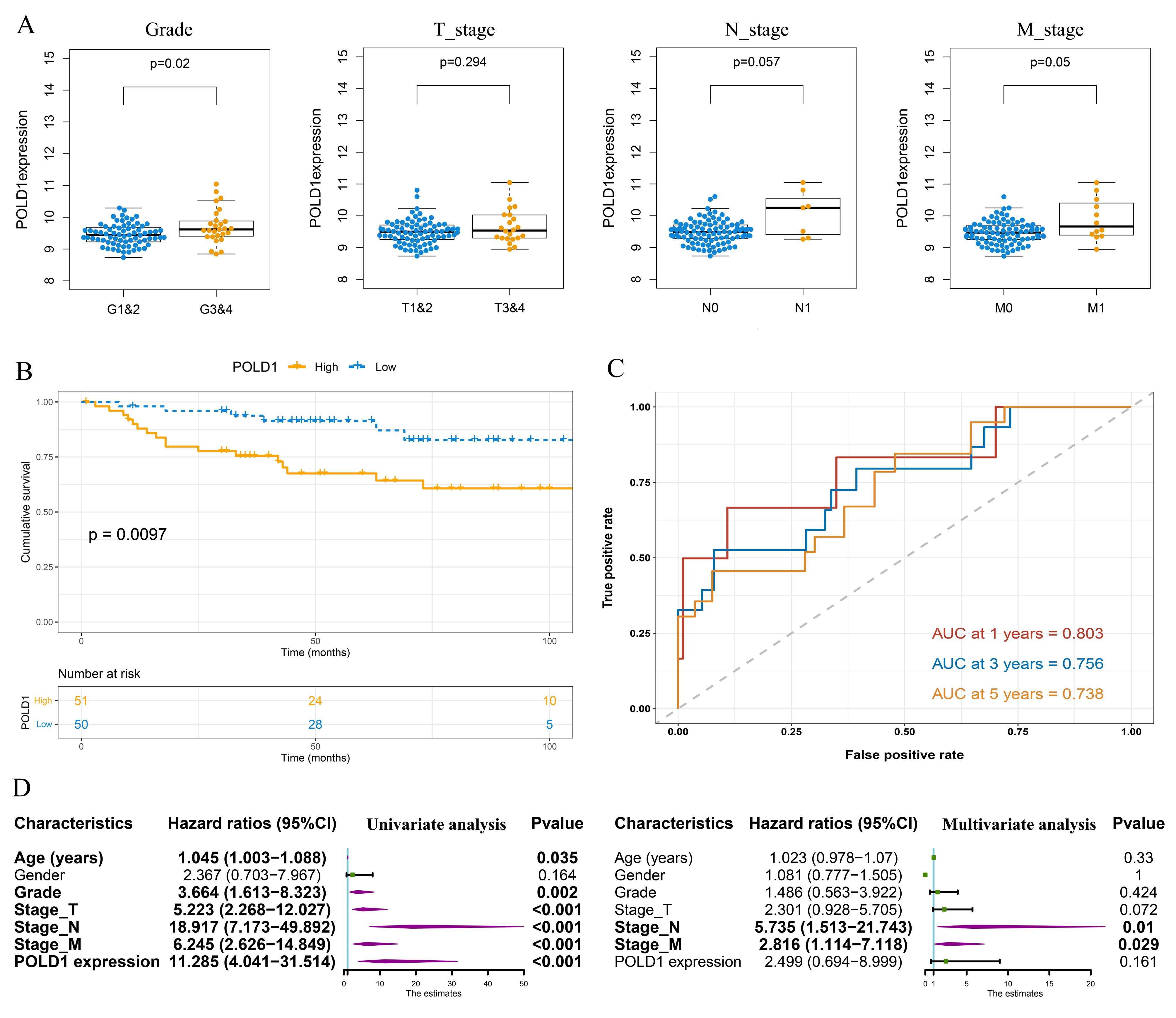

Supplement: Supplementary file 1 [file ijms-24-06849-s001.zip › Supplementary Figure 1.tif]

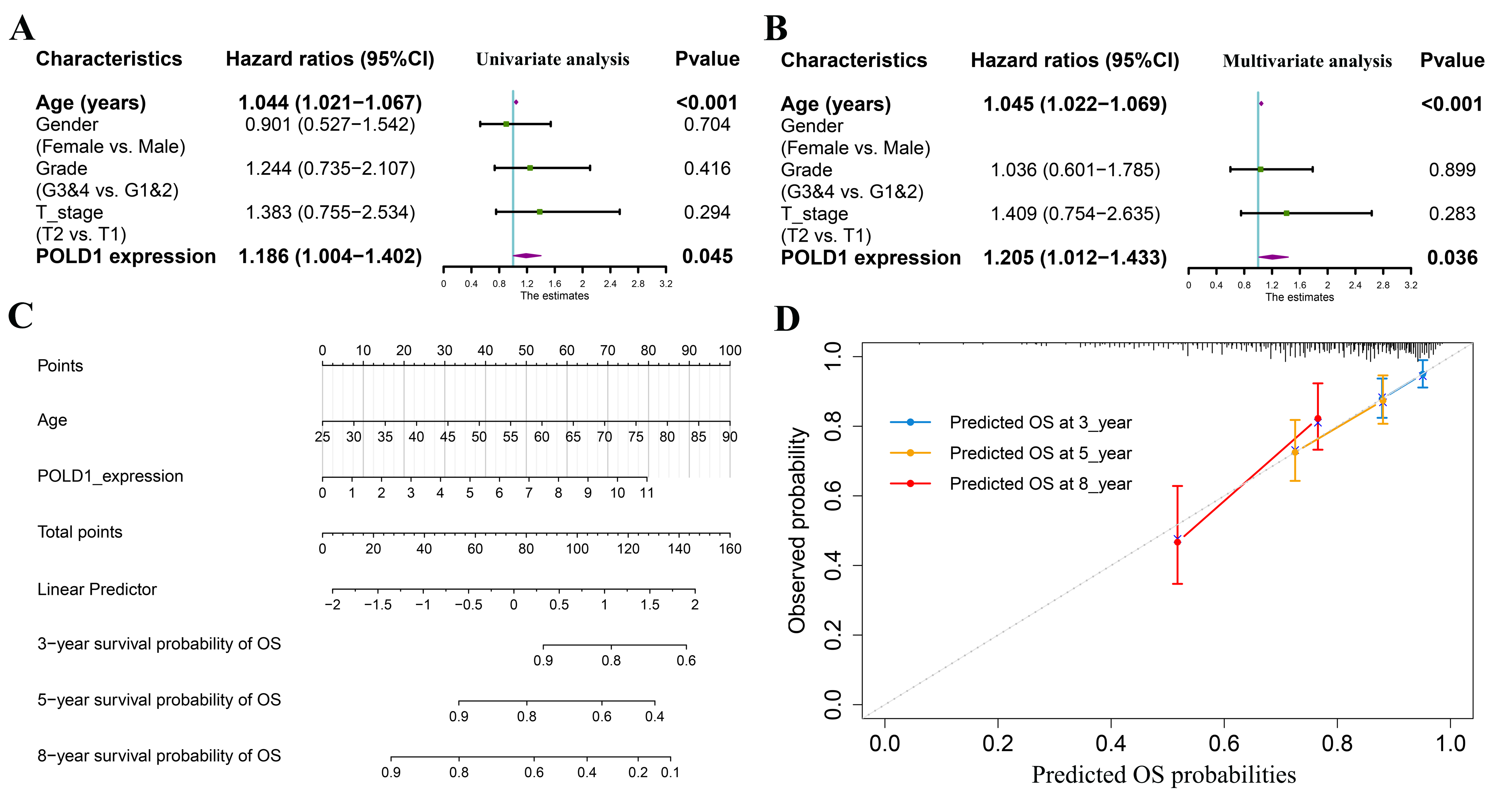

Supplement: Supplementary file 1 [file ijms-24-06849-s001.zip › Supplementary Figure 2.tif]

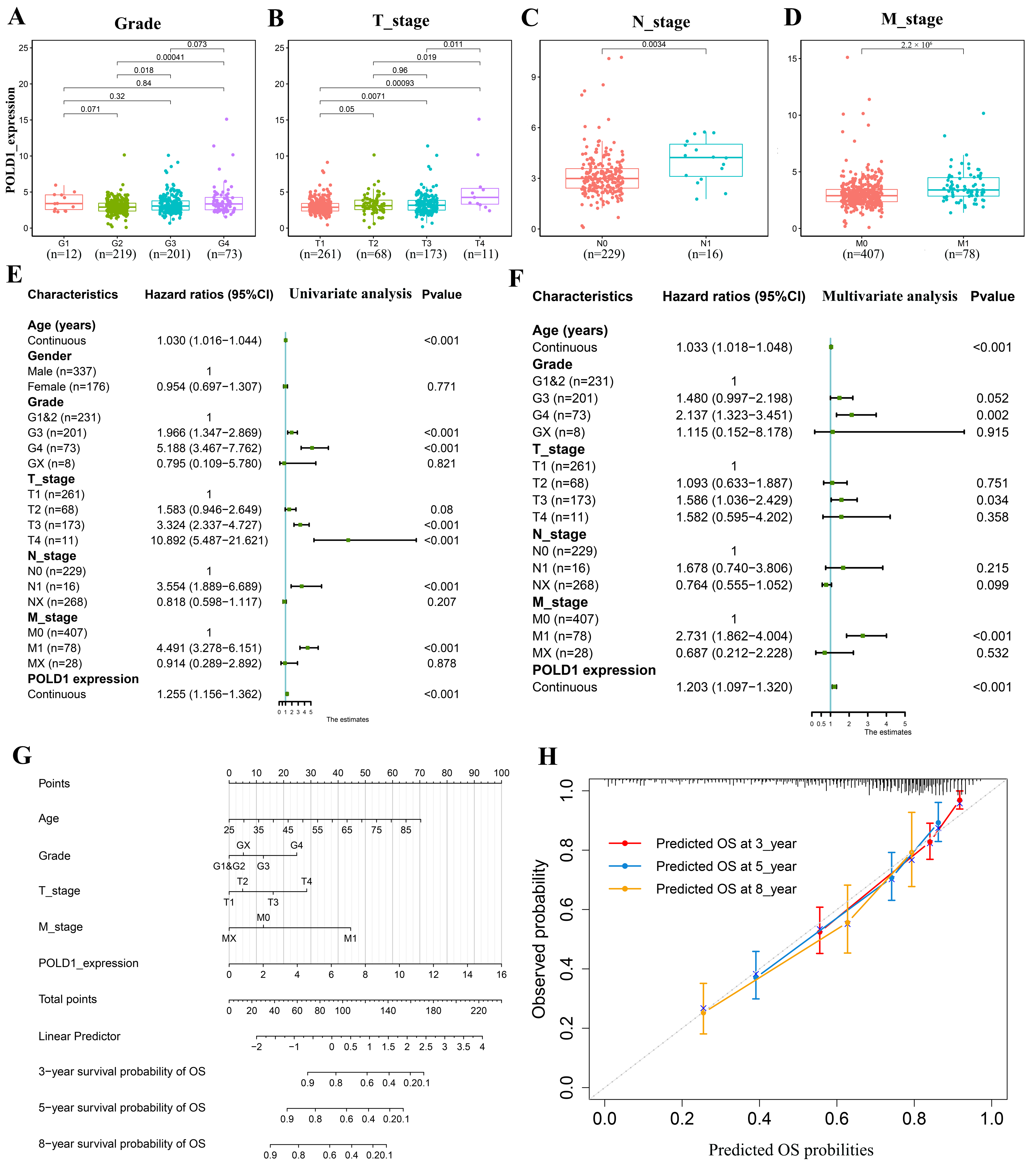

Supplement: Supplementary file 1 [file ijms-24-06849-s001.zip › Supplementary Figure 3.tif]

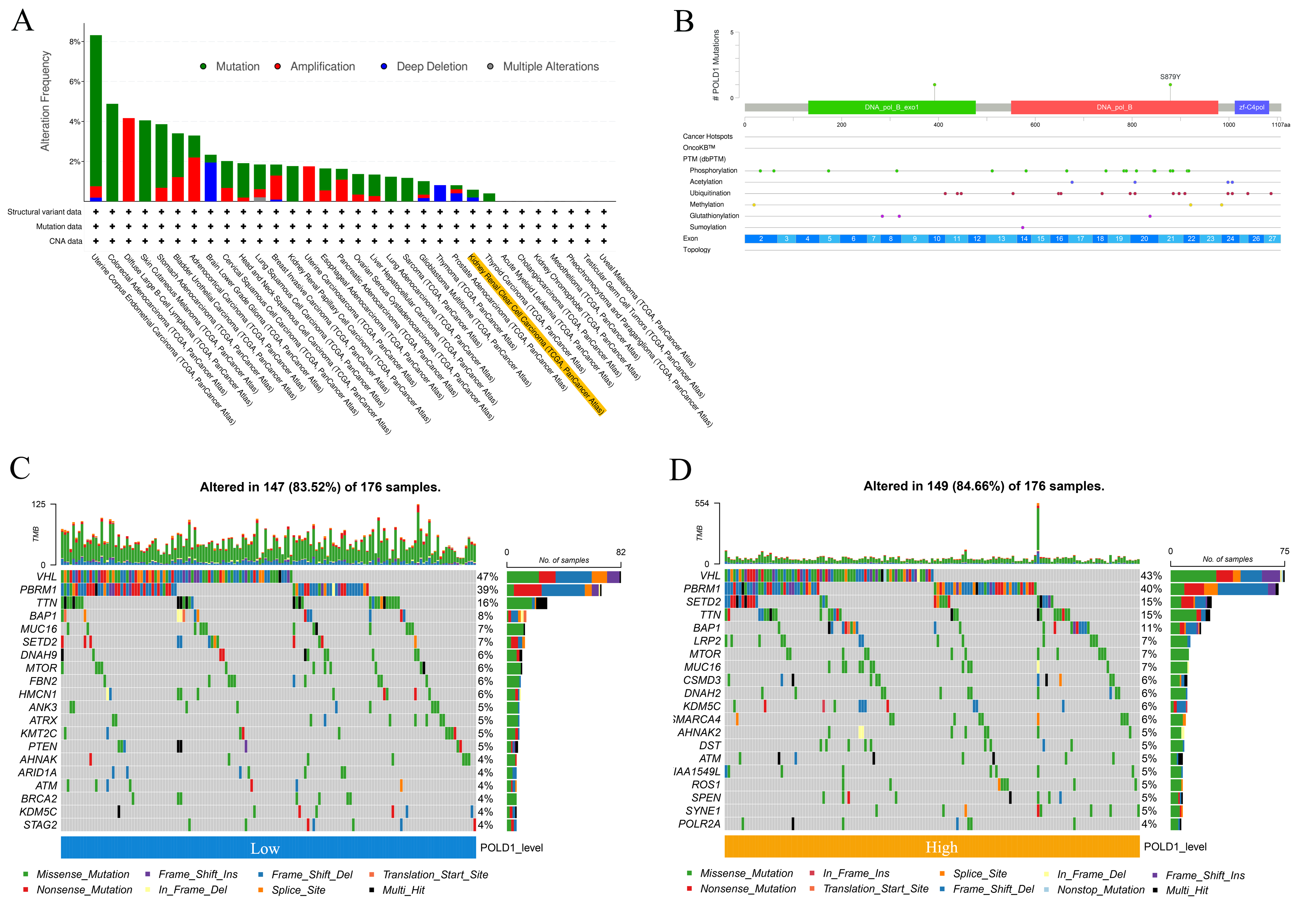

Supplement: Supplementary file 1 [file ijms-24-06849-s001.zip › Supplementary Figure 4.tif]

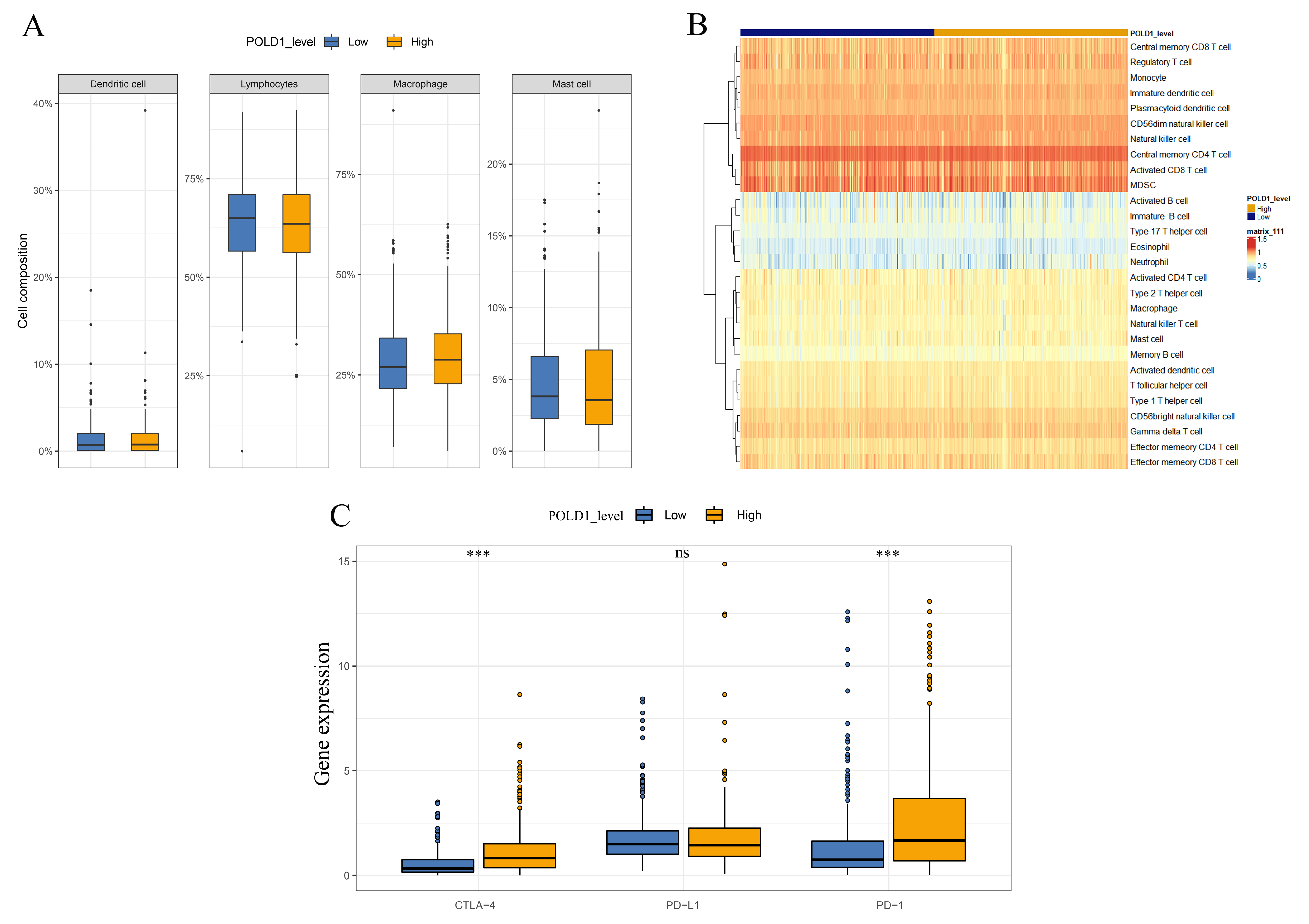

Supplement: Supplementary file 1 [file ijms-24-06849-s001.zip › Supplementary Figure 5.tif]
